# Use of the gonadal tissue of the sea urchin *Paracentrotus lividus* as a target for environmental contamination by trace metals

**DOI:** 10.1007/s11356-023-28472-2

**Published:** 2023-07-16

**Authors:** Monique S. Sarly, Carmen A. Pedro, Catarina S. Bruno, Andreia Raposo, Helenita C. Quadros, Ana Pombo, Sílvia C. Gonçalves

**Affiliations:** 1 MARE – Marine and Environmental Sciences Centre, ESTM - School of Tourism and Maritime Technology, Polytechnic of Leiria, 2520-641 Peniche, Portugal; 2 Gonçalo Moniz Institute - Oswaldo Cruz Foundation (Fiocruz), Salvador, 40296-710 Brazil; 3 MARE – Marine and Environmental Sciences Centre, Department of Life Sciences, Faculty of Sciences and Technology, University of Coimbra, 3004-517 Coimbra, Portugal

**Keywords:** *Paracentrotus lividus*, Histopathological biomarkers, Trace metals, Environmental impact, Coastal waters, Oil spill

## Abstract

Many environmental monitoring works have been carried out using biomarkers as a tool to identify the effects of oil contamination on marine organisms; however, only a few studies have used sea urchin gonadal tissue for this purpose. Within this context, the present work aimed to understand the impact of an oil spill, proposing the use of sea urchin gonadal tissue as a biomarker for environmental contamination by trace metals in the species *Paracentrotus lividus.* Biometric analysis, quantification analyses of the elements Cd, Pb, Ni, Fe, Mn, Zn, and Cu, as well as histopathological evaluations were performed in gonads of *P. lividus* collected from an area affected by hydrocarbons, named as impacted shore (IS) and an area not affected, named reference shore (RS). The results showed that carapace diameter (DC), total wet weight (WW), and Cd concentrations in the gonads were significantly influenced by the interaction between the rocky shores of origin, the months of sampling, and by the sex of the individuals. Moreover, from July until September, the levels of Zn and Cd were significantly lower in male than in female gonads. In July (the month of the oil spill), the indexes of histopathological alterations (IHPA) of membrane dilation were significantly higher in individuals from the IS, compared to the individuals from the RS. In addition, there were significant correlations between biometric variables (wet weight, diameter of carapace, gonadal weight, and gonadosomatic index) and the elements Cd, Cu, Ni, and Mn concentrations. Lastly, a delay in the gametogenic cycle of the sea urchins from IS was also observed. Taken together, these findings suggest that direct exposure to trace metals induces histopathological lesions in *P. lividus*’ gonads and affects its reproductive cycle.

**Graphical Abstract:**

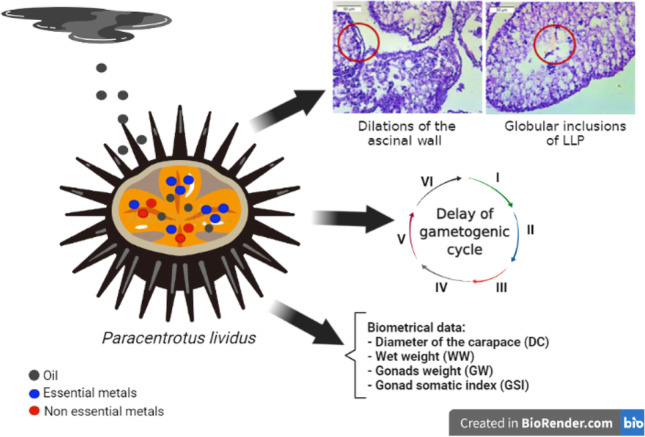

## Introduction

The increase of human populations and the consequent increase in anthropological activities have exposed coastal marine waters to a variety of contaminants (Odeku and Paulos 2017; Zaynab et al. 2022). The most frequent causes of contamination are associated to plastic debris, sewage and industrial effluents, oil spills, and “no-point sources” (inaccurate sources of pollution that do not originate from a single discrete source and issues widely pervasive environmental elements) (Odeku and Paulos 2017). The accumulation of contaminants over a long period in the coastal marine environment could be quite harmful resulting in devastating consequences for marine life and habitats on which marine organisms depend on (Elliott 2003; Mearns et al. 2020).

Among the environmental problems that strongly affect the health of oceans, pollution by oil is one of the most common (Rout and Sharma 2013). Crude oil is composed by a complex mixture of organic compounds, basically consisting of polycyclic aromatic hydrocarbons (PAHs), some heterocyclic compounds, and some trace metals (Dupuis and Ucan-Marin 2015). The most numerous elements in crude oil are carbon and hydrogen, ranging from 82 to 87% and 12 to 15%, respectively (Khuhawar et al. 2012). Subsequently to these elements, sulfur, oxygen, and nitrogen are also found. Sulfur contents range between 0.05 and 5%; oxygen is typically measured at less than 2%; and nitrogen contents are less than 0.1%. The other elements are found at the trace level or ultra-trace level; that is, between 0.01 and 0.1% or below 0.01% (such as nickel, iron, copper, zinc, manganese, cadmium, magnesium, and aluminum) (Yang et al. 2018).

The “trace metals” term refers to the elements with an atomic number greater than 20 (Ali and Khan 2018). Currently, this term admits a connotation of toxic and harmful chemicals (i.e., toxic and bio-accumulative), being some of them also endocrine disruptors and carcinogens (Kibria et al. 2016). These elements can exist in aquatic ecosystems, in organisms in a wide variety of chemical forms, and in combination with other materials. Among the metals of greatest concern in aquatic systems are cadmium (Cd), copper (Cu), lead (Pb), and zinc (Zn), which are toxic to organisms above specified threshold concentrations, although they may be essential for metabolism at lower concentrations (Kibria et al. 2010). The absorption or direct contact of some metals with the tissues of organisms can lead to damage and disturbance of cell membranes and cellular functions at the molecular level. If the concentration and duration of exposure to these toxic contaminants are high enough, the organisms can undergo potential physiological damage, with structural changes at the cellular level that can culminate in death (Soualili et al. 2008; Vikas and Dwarakish 2015; Lu et al. 2018). Therefore, monitoring potentially dangerous trace metals in crude oils or their derivatives which can harm the environment has become increasingly necessary.

Many environmental quality evaluation works have been carried out using biomarkers as a tool to monitor contamination from oil spills (Cunha et al. 2005; Anderson and Lee 2006; Duan et al. 2018; Walter et al. 2019; Wang et al. 2019). Biomarkers are represented by existing changes on cells, tissues, and organs in organisms that act as an effective device, considering that they can anticipate the detection of possible important disturbances at higher levels of organization (Schettino et al. 2012; Huggett 2018). These changes or disturbances can be associated with exposure to xenobiotics in contaminated environments and can be used to indicate toxic effects of these contaminants on target organs, reveal paths of exposure, besides reflecting the bioavailability of toxic substances in the environment (Huggett 2018; Gardner 1993).

Embryonic and larval stages of sea urchin development have been commonly used as biomarkers of the presence of environmental contaminants (Chiarelli et al. 2019; Gambardella et al. 2021; Kobayashi and Okamura, 2004; Migliaccio et al. 2015). However, adult sea urchins may represent an important source of environmental information from specific regions. As an animal that generally has restricted locomotor capacity and lives in coastal shores attached to the substrate (Hereu, 2005; Pearse 2006; Dumont et al. 2007; Scanu et al. 2015), it may be more exposed to pollutants, for example from soil leaching. Thus, they can act as biomarkers, revealing contaminants present in very specific regions. When living in or exposed to water contaminated by some pollutants, sea urchins can present alterations associated with their reproductive, physiological, and morphological aspects, which vary according to the levels of pollution (Danis et al. 2005; Soualili et al. 2008; Schäfer and Köhler 2009; Chiarelli and Roccheri 2014). Especially the variations in cell components and histopathological biomarkers identified in their reproductive organs can be used to expand the understanding of the harmful effects that environmental pollutants can trigger in the reproduction process of these marine organisms (Au 2004; Dietrich and Krieger 2009; AnvariFar et al. 2018).

The presence of contamination with oil derivates was already correlated with histopathological lesions in the gonads of sea urchins by Schäfer et al. (2011) and Vaschenko et al. (2001). The most frequently investigated histopathological criteria in these animals are degenerative germ cell processes, such as oocyte atresia and gamete resorption, and the process of lipid and protein peroxidation (Anderson 1968; Khristoforova et al. 1984; Vaschenko et al. 2001). Oocyte atresia and gamete resorption is an event that occurs naturally in the gonads; however, it can become pathological after a situation of exposure to pollutants (Blazer 2002; Dietrich and Krieger 2009). Additionally, the process of lipid and protein peroxidation gives rise to the fluorescent pigment called lipofuscin. Such injuries normally accumulate in tissues and cells as a result of environmental stress (Vaschenko et al. 2012). Therefore, the availability of practical and functional methodological tools, such as histopathological biomarkers, can enable a safe and reliable diagnosis of the effects of exposure of aquatic organisms to contaminants in the event of environmental accidents.

The sea urchin *P. lividus* has been qualified as an excellent bioindicator species of contaminants at the marine environment (Warnau et al. 1995a; Soualili et al. 2008; Rocha et al. 2018; Parra-Luna et al. 2020). This species belongs to the Parechinidae family and lives in rocky substrates and in seagrass meadows, from shallow water to about 20-m depth (Tenuzzo et al. 2012). These organisms feed mainly on aquatic vascular plants and algae but may also feed on debris in the water column, playing an important role in the structure and dynamics of their coastal ecosystems. They have a wide distribution in the rocky bottoms of western Europe, from Ireland to Portugal, south of Morocco through the Mediterranean Sea and part of the northwest African coast, both in unpolluted and polluted areas (Bayed et al. 2005; Boudouresque and Verlaque 2001).

In July 2017, an accidental spill of 3 t of hydrocarbons occurred at Abalo’s beach, on the coast of Peniche (Portugal). The fuel oil spill was originated in the system of feeding a boiler of an industrial company, based in the industrial area of the city and temporarily exposed this stretch of coast to those pollutants and to their derivatives, namely trace metals. Within the panorama presented, the main objective of the present work is to understand the impact of such an oil spill, proposing the use of sea urchin gonadal tissue as a biomarker for environmental contamination by trace metals in the species *Paracentrotus lividus*.

## Material and methods

### Collection of sea urchins

**Fig. 1:**
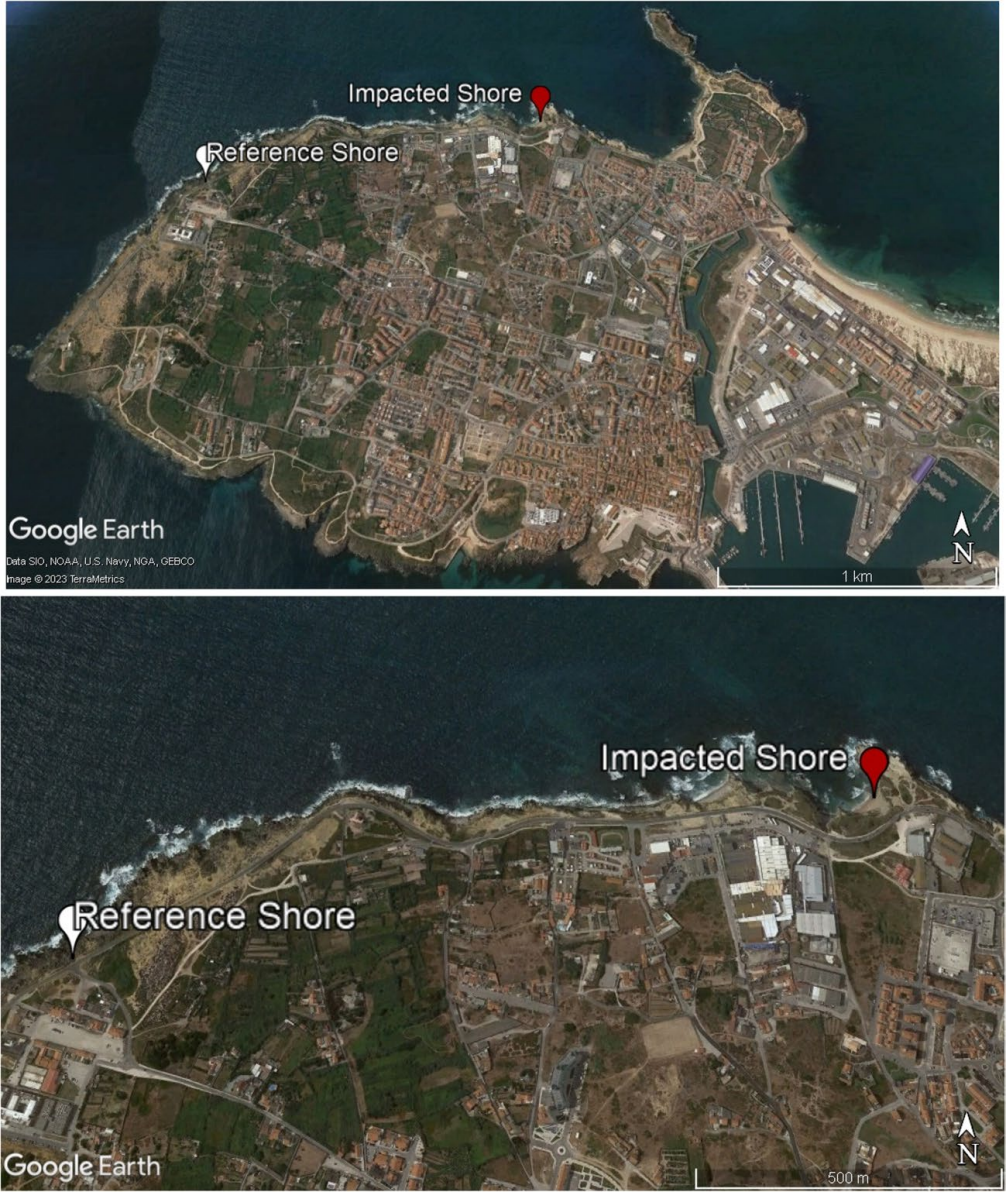
Map of Peniche (Portugal) with the indication of the study sites (impacted Shore, 39°22′12.69″N; 009°23′7.07″W; reference shore, 39°22′02.4″N; 9°24′08.07″W) where the specimens of *Paracentrotus lividus* were sampled

On July 6, 2017, a spill of 3 t of naphtha was recorded on the coast of Peniche (Portugal), due to a rupture of a fuel oil pipeline in the tanks that feed the boilers of a manufacturing facility. In this context, a total of 180 individuals were collected randomly during low tide on two rocky shores in the coastal area of Peniche, from July (a few days after the oil spill) to September in 2017, with 30 individuals taken monthly from each rocky shore. The first sampling area, called Abalo’s Beach (impacted shore — IS, 39°22′12.69″N; 009°23′7.07″W, *n* = 90), is located next to an area with several industrial activities and companies with intense movement, and was the central point where the fuel oil spill happened (Fig. 1). Despite the rapid intervention by the competent authorities (including APA, the Portuguese Environment Agency) to contain its impact, the access to Abalo’s Beach was blocked for 19 days and collecting samples from the PAHs for analysis was not possible. The second area, located 1.5 km to the west of IS and hereafter called reference shore (RS, 39°22′02.4″N; 9°24′08.07″W) was not directly affected by the spill. It is used as a control area for comparison in the present study (Fig. 1).

After collection, the animals were transported to the aquaculture laboratory of MARE-Polytechnic of Leiria — Marine and Environmental Sciences Centre, campus of the Polytechnic of Leiria, inside buckets with local marine water. In the laboratory, the horizontal diameter of the carapace (DC; ± 0.1-mm accuracy) of each specimen was measured with the help of a caliper (±1-mm accuracy) (Lindner, Arnstorf, Germany). Also, the total wet weight (WW; ± 0.01 g) and the gonads weight (GW; ± 0.01 g) of each individual were obtained using an analytical balance (AE ADAM PGL 3002, Milton Keynes, England). Based on these information, it was possible to calculate the gonad somatic index (GSI) from the following formula (Marsh et al. 2013): GSI (%) = gonad weight/wet weight × 100.

All 180 individuals collected were histologically analyzed, with one of the five gonads in each individual removed, fixed and preserved in 4% buffered formalin for further histological analysis, according to James et al. 2018. The other four gonads were stored in individual tubes at −80 °C and later lyophilized for metal concentration analyses.

### Metal concentration analyses

To determine the concentration of the metals cadmium (Cd), lead (Pb), nickel (Ni), iron (Fe), manganese (Mn), zinc (Zn), and copper (Cu), sixteen sea urchins (eight males and eight females) from each sampling month and from each rocky shore, were randomly selected. After that, approximately 100 mg of dried *P. lividus* gonadal samples were accurately weighed into a Teflon microwave digestion tube and digested using 10 ml of HNO_3_ (69.6%, AnalaR NORMAPUR, VWR pro lab chemicals, France) in a high-performance microwave digestion system (Milestone connect, MA182-001 Ethos up, Italy). The concentrations of non-essential (Cd, Ni, and Pb) and essential (Fe, Mn, Zn, and Cu) metals in the samples of the gonads were assessed in triplicate and determined by Atomic Absorption Spectrometry (AAS) (Thermo Scientific™ iCE™ 3500, Thermo Unicam, Portugal). The results were expressed as milligrams per kilogram of dry weight. In all quantifications, 1% HNO_3_ was used as background and subtracted. The results were expressed by mean values ± standard deviations (Pedro et al. 2013). To verify the accuracy of the method, a known amount of analyte was added to the natural test sample matrix, and its response was measured (recovered) in the assay, compared to an identical elevation in the standard diluent. The objective of this procedure was to evaluate if the methodology used in this work was valid, thus allowing the repeatability of the analysis.

### Histological analysis

For histological analysis, the gonads were fixed in 4% buffered formalin for 24 h and then inserted into a solution of 70% ethanol also for 24 h. Subsequently, the gonads were processed in a tissue processor (Leica TP 1020, Nussloch, Germany), in which they passed through different ethanol solutions with increasing concentrations (dehydration step). After that, the gonads were clarified, in order to remove the dehydrating agent and replacing it with a liquid miscible (xylol) with the impregnation medium (liquid paraffin). In the last stage of the processor, the gonads were embedded in paraffin medium at 60 °C and then transformed in solid blocks of paraffin. Thereafter, the microtomy of the blocks was performed on a Rotary Microtome (Accu-Cut® SRM™ 200) obtaining 5-μm thickness sections. The sections were dried and preserved in a kiln at 37 °C (Binder, Tuttlingen, Germany) for 24 h until they were subjected to the staining technique. The sections were stained with hematoxylin and eosin (H&E). After staining, the slides were assembled using Coverquick 2000 Path® assembling medium (San Francisco, USA) and then dried for 24 h at room temperature. Finally, the slides were observed under a composite optical microscope (Leica DM 2000 LED, *Wetzlar*, Germany) and photographed using a microscope camera (Leica® MC170 5MP HD), combined with LAS V4.4.0 software (Leica Application Suite) for monitor display (Leica Microsystems GmbH). From the application of the staining technique, it was possible to determine the sex of the individuals and to characterize the different stages of gametogenesis, according to Byrne (1990) and Spirlet et al. (1998). The stages were identified based on the following characteristics:

(i) Stage I — early phase: the gonad lumen is completely filled with nutritive phagocytes (NP), defined as non-germ accessory cells, which can vary in size and color. It is possible to observe the presence of primary oocytes along the ovarian/testicular ascinal wall, which in turn is covered by a thin basophilic layer;
(ii) Stage II — growth: it is from this stage that cell growth gradually begins. The lumen of the gonad is occupied by nourishing phagocytes and the pre-yolk oocytes, which are still along the ascending wall of the ovary/testis and begin to grow, as they absorb nutrients supplied by NPs. The female gamete assumes the typical form of vitellogenin oocyte (OV). In males, sperm begin to protrude centrally and the basophilic layer increases;
(iii) Stage III — pre-maturation: there is a reduction in the presence of NPs and an increase in oocytes in number and size. It is still possible to observe pre-yolk oocytes attached to the gonadal tissue wall, surrounded by nutritive phagocytes (NPs). As vitellogenesis occurs, there is also a migration of mature oocytes to the center of the acino. At this stage vitellogenesis is a continuous process and oocytes in all stages are present;
(iv) Stage IV — maturation: a large number of mature oocytes (90 μm in diameter) occupy the lumen of the acini. Eventually, nutritive phagocytes can be observed forming a very thin layer near the ascinal wall, as well as some small vitellogenin oocytes (10 to 60-μm diameter) present in the ascinal wall, indicating that the vitellogenin process has not yet been completed. In males, the mature testes are full of sperm and the nutritive phagocytes are limited to the ascinal wall;
(v) Stage V — posture: at this stage, the acini contracts and is emptied; however remnant mature oocytes (OR) can eventually be observed, which will later be reabsorbed by nutritive phagocytes (NPs). In females there are spaces between the unreleased oocytes. In males, this stage has a very similar aspect to stage IV, differing only by the presence of spaces in the lumen and the smaller amount of sperm. Also, the ascinal wall looks thin and sperm may be present in the gonoduct;
(vi) Stage VI — spent stage: at this stage, the ovaries have thin ascinal walls, lose their internal structure leading to disorganization, and it is possible to observe mature oocytes and pre-vitelline oocytes, which have detached from the ascinal wall. Any oocytes present in the ovary at this stage will probably be reabsorbed. One may also observe a meshwork of nutritive phagocytes around the periphery that may have begun to sequester reserves for the next oogenic cycle. In males, thin ascinal walls and a meshwork of nutritive phagocytes are observed on the periphery of the testis, as well as spaces created by the absence of sperm.

#### Histopathological analysis

During the identification of the gametogenic stages of the sea urchins, we did careful observations to detect the presence of anomalies, lesions or other histopathological changes in their appearance that could be indicative of an environmental pollution scenario. Thus, an index of histopathological alterations (IHPA) was determined by analyzing the following gonad lesions: oocyte resorption (IHPA reabs), dilation of acinal wall (IHPA dilat), accumulation of lipofuscin-like pigments (IHPA LLP), nutritive phagocyte hypertrophy (IHPA hyper), or atrophy (IHPA atro).

The histopathological lesions were identified according to Schäfer (2009) and Vaschenko et al. (2012). To verify the accumulation of lipofuscin-like pigments (IHPA LLP), the presence of high amounts of lipofuscin were sought in extracellular spaces and in the oocytes of some individuals, which are characterized by large globular inclusions of gold–yellow to brown–yellow color. In sequence, the identification of hypertrophy of nutritive phagocytes (IHPA hyper) was carried out by observing accumulations and expanded sizes of these cells due to the increase in the synthesis of their basic constituents and their volume. NP atrophy (IHPA atro), in turn, was found when smaller cells were identified as a result of the decreased nutrition, metabolism, and synthesis needed to renew their structures. Resorption of oocytes (IHPA reabs) was identified when a higher volume density of atretic oocytes was observed. Finally, dilation of acinal wall (IHPA dilat) was characterized when two epithelial layers were separated from each other. Often, this dilation is concomitant with an increased formation of collagen between the epithelial layers and an increase in basophilic material (Schäfer 2009; Vaschenko et al. 2012).

Based on the observations carried out, the gonad lesions were classified on a scale of zero (no lesions) to three (severe). To calculate the IHPA, the total score of the different pathologies observed in the gonads was divided by the number of individuals analyzed (Vaschenko et al. 2012).

### Statistical analysis

Prior to any statistical analysis, all data were tested for normality (test Kolmogorov-Smirnov) and homogeneity of variance (Levene’s test) and, when necessary, the data that did not meet these assumptions were transformed. When the transformations did not remove the heterogeneity, the analyses were performed on the untransformed data (whenever *n* ≥ 30), since analysis of variance is quite robust to departures from their assumptions (Underwood, 1997). Three-way ANOVA analyses were used to test the effects of the sampling stations, months of study, and the animals’ sex, as well as the interaction between the 3 factors, on biometric responses and gonadal lesions. The significant effects detected were then subjected to post hoc tests: (i) *Tukey HSD* to analyze the individual effects of the factors and (ii) Bonferroni tests to analyze the significant interactions between the factors. All these analyses were performed using the software IBM® SPSS® Statistics. Also, to test if the presence of metals on the gonads of *P. lividus* had any influence on their biological variables, Spearman correlations were performed between the bioaccumulated metals in the gonads and biometric data and the index of histopathological lesions through the Prism version 5.01 software (GraphPad Software, La Jolla, CA, USA). Differences with *p* values < 0.05 were considered significant. All data are presented as mean ± standard deviation (S.D.).

## Results

### Biometric data

The three-way ANOVA analysis revealed that the months of sampling and the rocky shores influenced some of the biometric variables while the sex of the individuals collected, as an isolated factor, had no statistical influence on the biometric responses (Table 1).

**Table 1:** Three-way ANOVA and post hoc tests results for the biometric characterization, the concentrations of Cd, Pb, Cu, Zn, Ni, Fe, and Mn, and the index of histopathological lesions in gonads of *Paracentrotus lividus* from 2 different sampling stations in the coast of Peniche (Portugal), from July to September of 2017, following an oil spill event

ANOVA	*df*	MS	*F*-statistic	*p*-value
Source of variation				
Gonadosomatic index				
Rocky shores	1	176.839	19.214	0.000
Months	2	83.421	9.064	0.000
Gonadal weight				
Months	2	7.715	10.798	0.000
Total wet weight				
Rocky shores × months × sexes	2	86.870	4.380	0.014
Carapace diameter				
Rocky shores × months × sexes	2	0.328	3.825	0.024
Pb				
Rocky shores	1	24.665	4.974	0.028
Cu				
Months	2	287.358	6.008	0.004
Zn				
Months × sexes	2	374373.416	3.975	0.022
Cd				
Rocky shores × months × sexes	2	0.989	3.244	0.044
IHPA Dilat				
Rocky shores	1	4.532	7.104	0.008
Months	2	17.528	27.478	0.000
IHPA Reabs				
Months	2	37.249	48.973	0.000
Sex	1	3.224	4.239	0.041
IHPA LLP				
Months	2	3.274	3.835	0.024
Sex	1	6.433	7.537	0.007
IHPA Hyper				
Months	2	3.904	3.062	0.049
IHPA Atro				
Sex	1	13.811	13.695	0.000
Post hoc tests				
Dependent variable and factors tested	Test	Condition		*p*-value
Gonadosomatic index				
Months	Tukey HSD	Comparison: July and September		0.000
Gonadal weight				
Months	Tukey HSD	Comparison: August and September		0.000
		July and September		0.000
Total wet weight				
Interaction: Rocky shores × months × sexes	Bonferroni	Comparison: July and August		0.025
		August and September		0.000
		July and September		0.008
Carapace diameter				
Interaction: Rocky shores × months × sexes	Bonferroni	Comparison: July and August		0.016
		August and September		0.000
Cu				
Months	Tukey HSD	Comparison: July and September		0.003
Cd				
Interaction: Rocky shores x months × sexes	Bonferroni	Comparison: August and September		0.031
		July and September		0.009
Post hoc tests				
Dependent variable and factors tested	Test	Condition		*p*-value
Zn				
Interaction: Months × sexes	Bonferroni	Comparison: July and September		0.014
IHPA Dilat				
Months	Tukey HSD	Comparison: July and August		0.000
		July and September		0.000
IHPA Reabs				
Months	Tukey HSD	Comparison: July and August		0.000
		August and September		0.000
		July and September		0.000
IHPA LLP				
Months	Tukey HSD	Comparison: July and September		0.006
IHPA Hyper				
Months	Tukey HSD	Comparison: July and August		0.0023

Only the variables that presented significant results were represented (*ρ*-value < 0.05). The effects of different sampling stations (impacted shore and reference shore), the gender of individuals (males and females) and different months (July, August, and September) were considered as factors

*df* degrees of freedom, MS – mean square. *IHPA Dilat* index of membrane dilation, *IHPA Reabs* index of sex cell resorption, *IHPA LLP* index of lipofuscin-like pigments accumulation, *IHPA Hyper* index of hypertrophy in nutritive phagocytes, *IHPA Atro* index of atrophy in nutritive phagocytes

**Fig. 2:**
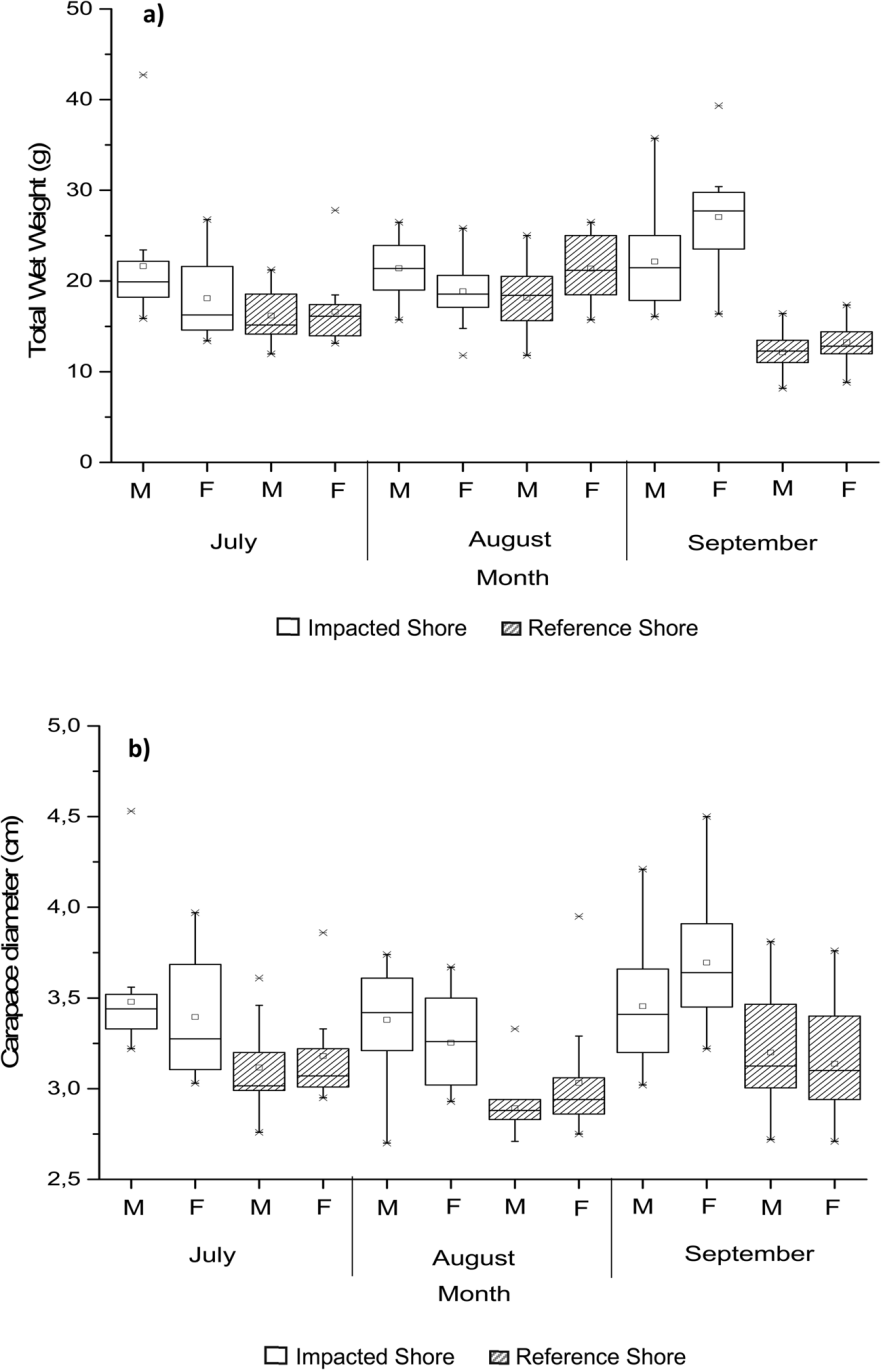
Total wet weight (WW) and diameter of the carapace (DC) average values of *Paracentrotus lividus* at the impacted shore (IS) and at the reference shore (RS) (Peniche, Portugal) collected in July, August, and September of 2017, after an oil spill event. (**a**) Total wet weight in grams (g); (**b**) carapace diameter in centimeters (cm)

The total wet weight (WW) of the sea urchins was significantly influenced by the interaction between the rocky shores of origin, the months of sampling, and by the sex of the individuals (*p* = 0.014; Table 1). In general, sea urchins from IS were significantly heavier than those from RS (WW: 21.08 ± 5.71 g *versus* WW: 15.26 ± 4.02 g, respectively; Table 1; Fig. 2a). In the month of September, the sea urchins presented the highest WW values (IS: 24.39 ± 5.86 g; RS: 16.71 ± 4.64 g); followed by July with intermediate values (IS: 19.94 ± 5.49 g; RS: 16.21 ± 3.59 g) and, lastly, August, in which the sea urchins presented the lowest WW (IS: 18.93 ± 4.26 g; RS: 12.86 ± 2.45 g). In all cases, the sea urchins collected at the IS showed higher mean WW values than the animals collected at RS (*Bonferroni test*; Table 1; Fig. 2a). As for the influence of sex, male individuals (IS: 19.91 g; RS: 15.04 g) collected in July at both sampling stations (IS and RS) were heavier than females (IS: 16.27g; RS: 15.95g). This trend was confirmed in the month of August for individuals collected in the IS (males heavier than females), while at RS females were significantly heavier, compared to males collected. Lastly, in September, female sea urchins were heavier than male sea urchins (*p* = 0.014; Table 1; Fig. 2a).

The diameter of the carapace of the sea urchins (DC) collected in this study ranged from 2.97 to 3.59 cm (observed in August at RS and in September at IS, respectively). Similar to what was observed for the WW data, the three-way ANOVA revealed that the DC of the individuals was significantly influenced by the interaction between the rocky shores, the sex of the individuals, and the months of sampling. Primarily, the organisms collected at IS were significantly higher than those from RS (DC: 3.45 ± 0.33 cm versus DC: 3.09 ± 0.28 cm, respectively; Table 1; Fig. 2b). In parallel to this, it was observed that the sea urchins with the highest values were recorded in September (IS: 3.59 ± 0.35 cm; RS: 3.16 ± 0.30 cm), while the smallest values were observed in August (IS: 3.32 ± 0.28 cm; RS: 2.97 ± 0.27 cm), being statistically different from each other (*Tukey HSD test*; *p* < 0.05). The month of July presented intermediate values which were statistically different from August (*Tukey HSD test*; *p* < 0.05), but not from September (*Bonferroni test*; Table 1) (Fig. 2b).

In July at both sampling stations, male organisms have higher mean values of DC (IS: 3.44 ± 0.29 cm; RS: 3.12 ± 0.25cm) than females (IS: 3.28 ± 0.35 cm; RS: 3.07 ± 0.27 cm). Likewise, in August at IS, male individuals were higher than the female sea urchins collected (male: 3.42 ± 0.29 cm and female: 3.26 ± 0.26 cm). In RS, still in this month, the opposite was observed, with females presenting highest carapace diameter values (male: 2.88 ± 0.23 cm and female: 2.94 ± 0.28 cm). Finally, in September, the reverse pattern was observed. In the IS, the females presented more expressive mean DC values (3.64 ± 0.35 cm), compared to the males (3.41 ± 0.33 cm); while in RS, male sea urchins (3.13 ± 0.33 cm) were higher than females (3.10 ± 0.29 cm).

**Fig. 3:**
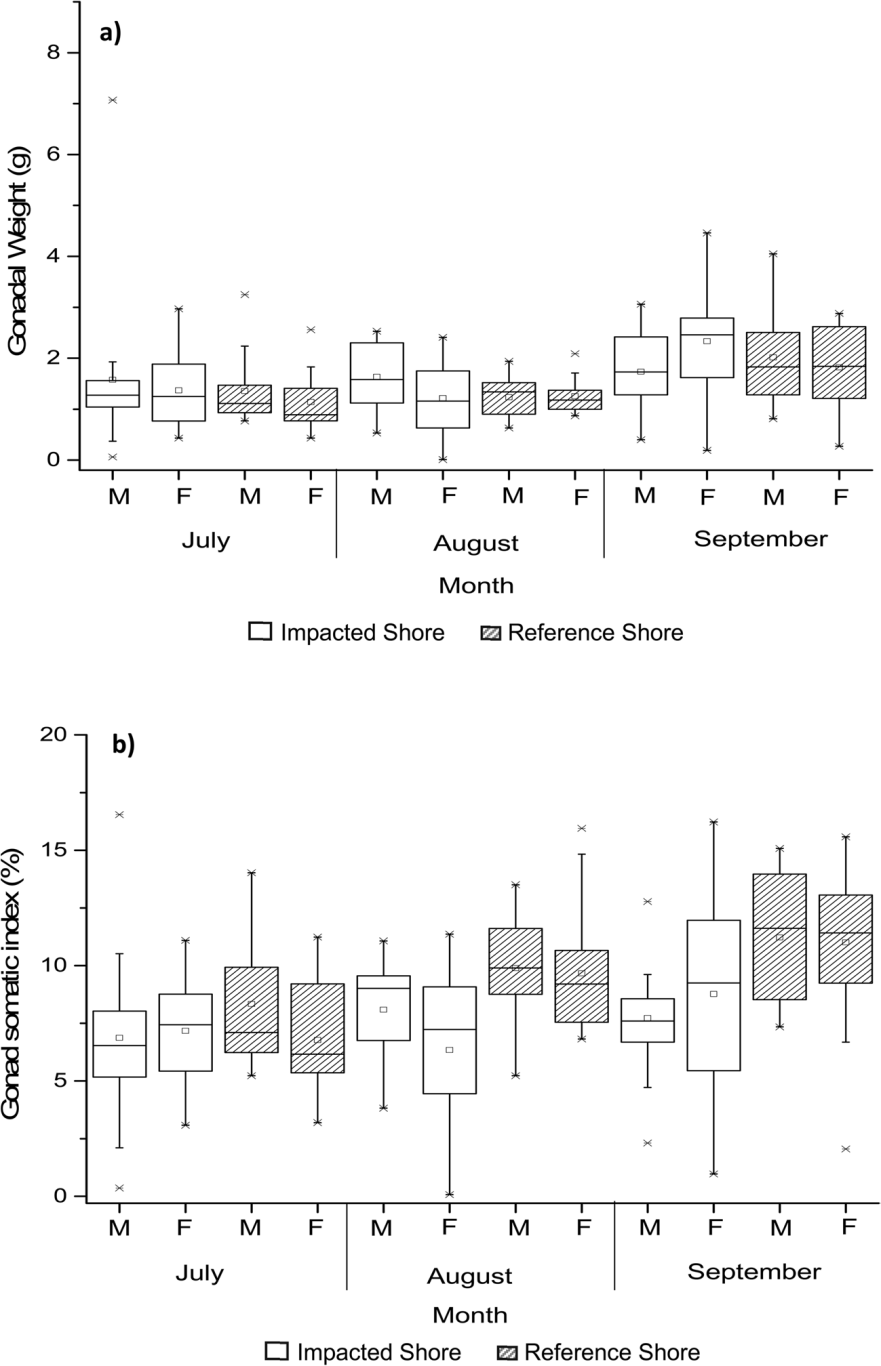
Gonadal weight (GW) and gonadosomatic index (GSI) average values of *Paracentrotus lividus* at the impacted shore (IS) and at the reference shore (RS) (Peniche, Portugal) collected in July, August, and September of 2017, after an oil spill event. (**a**) Gonadal weight (GW) in grams (g); (**b**) gonadosomatic index (GSI) in percentage (%)

Gonadal weight (GW) values did not show significant differences between the studied sampling sites or between sexes. On the other hand, over the months the values varied statistically (Table 1). In September, the GW of sea urchins reached the highest values (IS: 2.10 ± 1.05 g; RS: 1.90 ± 0.83 g), being statistically different from July (IS: 1.49 ± 1.23 g; RS: 1.26 ± 0.63 g) and August (IS: 1.43 ± 0.71 g; RS: 1.25 ± 0.35g) (*Tukey HSD test*; *p* = 0.000; Fig. 3a).

Finally, with regard to the gonadosomatic index (GSI) values, individuals collected at the IS had significantly lower GSI values (7.49 ± 0.72%) than individuals collected at the RS (9.50 ± 0.28%) (Fig. 3b). Among the studied months, the highest records of GSI were observed in September (IS: 8.32 ± 3.59%; RS: 11.09 ± 3.07%), being statistically different from the month of July, when the lowest values were recorded (IS: 6.99 ± 3.27%; RS: 6.99 ± 3.27%). The values observed in August were not statistically different from those observed in July and September (*Tukey HSD test*; *p* > 0.05) (Table 1; Fig. 3b).

### Metals

**Fig. 4:**
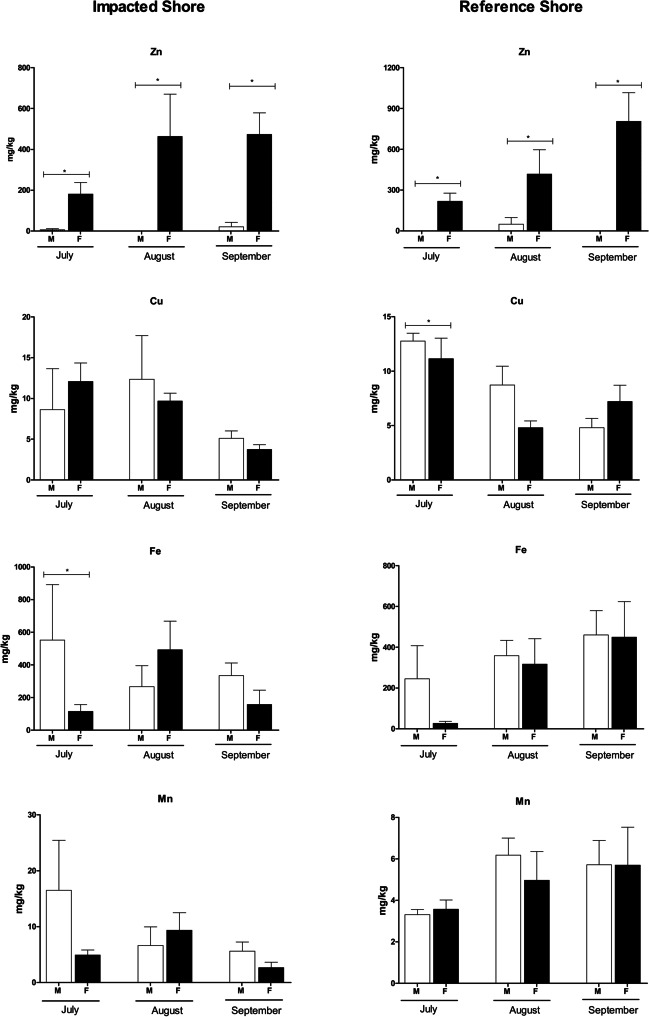
Concentrations of Cu, Zn, Fe, and Mn in the gonads of males and females of *Paracentrotus lividus* collected in the impacted shore and in the reference shore at Peniche (Portugal) in July, August, and September of 2017, following an oil spill event. All values were expressed as mean ± standard error. Significant differences (*p* ≤ 0.05) are presented with the symbol * (Tukey HSD test)

**Fig. 5:**
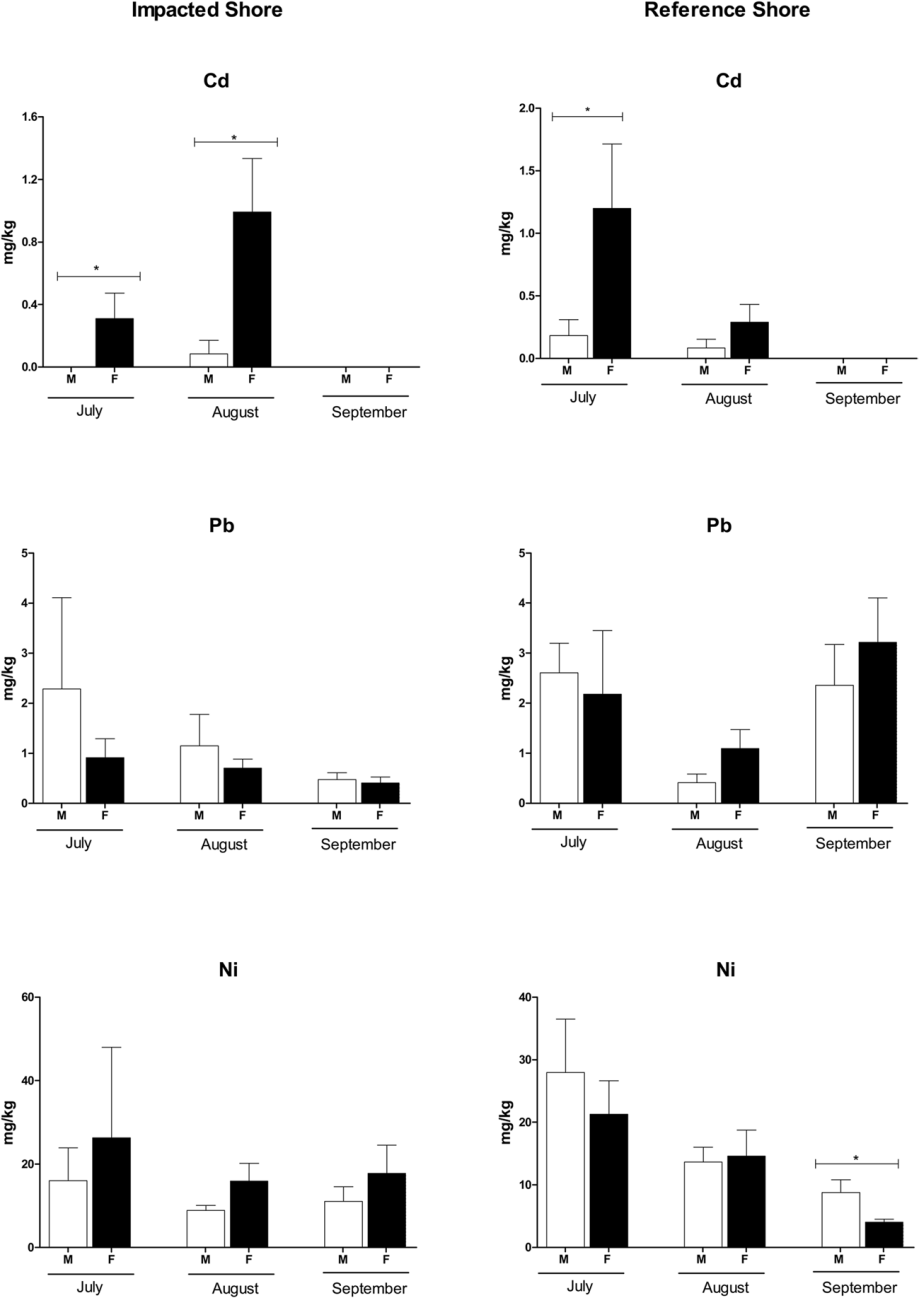
Concentrations of Cd, Pb, and Ni in the gonads of males and females of *Paracentrotus lividus* collected in the impacted shore and in the reference shore at Peniche (Portugal) in July, August, and September of 2017, following an oil spill event. All values were expressed as mean ± standard error. Significant differences (*p* ≤ 0.05) are presented with the symbol * (Tukey HSD test)

Concentrations of the seven metals under analysis were detected in *P. lividus* gonads from the two sampling sites during almost the entire period of study (July, August, and September as shown in Figs. 4 and 5). Among the essential metals studied in this research, Zn and Fe were the elements which exhibited the highest average concentrations (402.624 ± 145.80 mg/kg and 454.70 ± 101.99 mg/kg, respectively), registered in September at the RS. By comparison, the lowest concentrations of both metals were found in individuals with average concentrations of 93.14 ± 35.75 mg/kg for Zn at the IS and 136.09 ± 83.17 mg/kg for Fe at the RS, both in July. Regarding to the non-essential metals, the lowest concentrations were observed for Cd, which was not detected in any of the sampling stations in the month of September, and Pb with average concentrations of 0.43 ± 0.09 mg/kg in September at IS. The highest concentrations of these metals, in contrast, were recorded in July at RS for Cd (0.69 ± 0.29 mg/kg) and in September at RS for Pb (2.77 ± 0.60 mg/kg).

The results from the three-way ANOVA showed that the sex of organisms had a statistical effect when combined with months and rocky shores on Cd concentrations. In the group of non-essential metals, Cd was the only element where the concentrations were statistically different between genders, with female gonads exhibiting higher concentrations (IS: July: 0.31 ± 0.16 mg/kg; August: 0.99 ± 0.34 mg/kg; September: < LOD | RS: July: 1.20 ± 0.52 mg/kg; August: 0.29 ± 0.14 mg/kg; September: < LOD) than male gonads (IS: July: < LOD; August: 0.09 ± 0.09 mg/kg; September: < LOD | RS: July: 0.18 ± 0.13 mg/kg; August: 0.08 ± 0.07 mg/kg; September: < LOD) (Table 1; Fig. 4). As for the essential metals group, Zn was the only one that presented statistical differences in the mean values found in males and females, with the concentrations of this element being significantly influenced by the interaction between the sampling months and the sex of the individuals. Zn exhibited a similar pattern to that observed for Cd, in which female gonads had higher mean concentrations (IS: July: 180.45 ± 57.13 mg/kg; August: 462.70 ± 207.62 mg/kg; September: 472.85 ± 105.81 mg/kg | RS: July: 217.25 ± 60.96; August: 417.02 ± 179.78 mg/kg; September: 805.28 ± 211.64 mg/kg) than those found in male gonads (IS: July: 5.82 ± 5.82 mg/kg; August: < LOD; September: 20.97 ± 20.97 | RS: July: < LOD; August: 49.20 ± 49.20 mg/kg; September: < LOD) (Table 1; Fig. 4).

The analyses also revealed that the concentrations of Cu presented significant variations during some months of study. The lowest values of Cu were obtained during September with 4.42 ± 2.20 mg/kg and 6.00 ± 3.57 mg/kg in sea urchins collected in IS and RS, respectively. In both locations, Cu concentrations were significantly more expressive during the month of July, reaching concentrations of 10.34 ± 10.81 mg/kg in IS, and of 11.95 ± 3.99 mg/kg at RS (*Tukey HSD test; p* = 0.003).

Among sampling stations, mean concentrations of most of the studied metals varied little, and no significant differences were observed, except for Pb. The three-way ANOVA revealed that the mean values found for Pb were significantly and mostly more expressive in sea urchins collected in RS (July: 2.37 ± 2.73 mg/kg and September: 2.77 ± 2.78 mg/kg) when compared to the mean values found in the individuals collected in IS (July: 1.47 ± 3.74 mg/kg and September: 0.43 ± 2.77 mg/kg) (Table 1; Fig. 5). Only in the month of August, this trend was not confirmed, since the sea urchins collected in IS had higher average concentrations of Pb (0.91 ± 1.17 mg/kg), compared to individuals collected in RS (0.74 ± 0.88 mg/kg).

### Histology

#### Gametogenic cycle

During the histological analysis, all stages of gametogenic development were observed in the sea urchins collected. The relative frequencies of the different stages of the gametogenic cycle of *P. lividus* revealed to be heterogeneous over the 3 months of study, being statistically different (*X*^2^ (10) = 65.618; *p* < 0.05), so that at least two and up to five gonadal stages could be described in the monthly samples.

In the month of July, it was found that individuals from IS were mostly in stage V (males = 44.44%; females = 33.33%), while those from RS were mostly in stage VI (males = 16.67%; females = 50%). In August, it was observed that most sea urchins were in stage V and VI in both sampling stations, except the males from RS, in which stage IV was not identified. On the other hand, in September most of the individuals were at the beginning of a new gametogenic cycle at both sampling stations (stage I: males = 61% and females = 70% at IS; males = 50% and females = 61% at RS). In that same month, it was noted the low incidence of individuals in stage II (Growth), which was only observed in female individuals in August at RS (females = 7.69%) (Fig. 7). It was also possible to verify in September, some individuals in the final stages of the gametogenic cycle (V and VI), while the presence of stage IV was only observed in male individuals at the IS.

The analysis of the gametogenic stages showed that in general, there was a synchronized reproductive pattern between sexes. However, in August, males at the RS showed to be mainly in the stages III and V (23.07% both stages) and females in stage VI (52.94%). Finally, the number of individuals in stage VI (post spawning) was higher both in July and in August, suggesting that the spawning peak had occurred in the previous months.

#### Identification of histopathological lesions

**Fig. 6:**
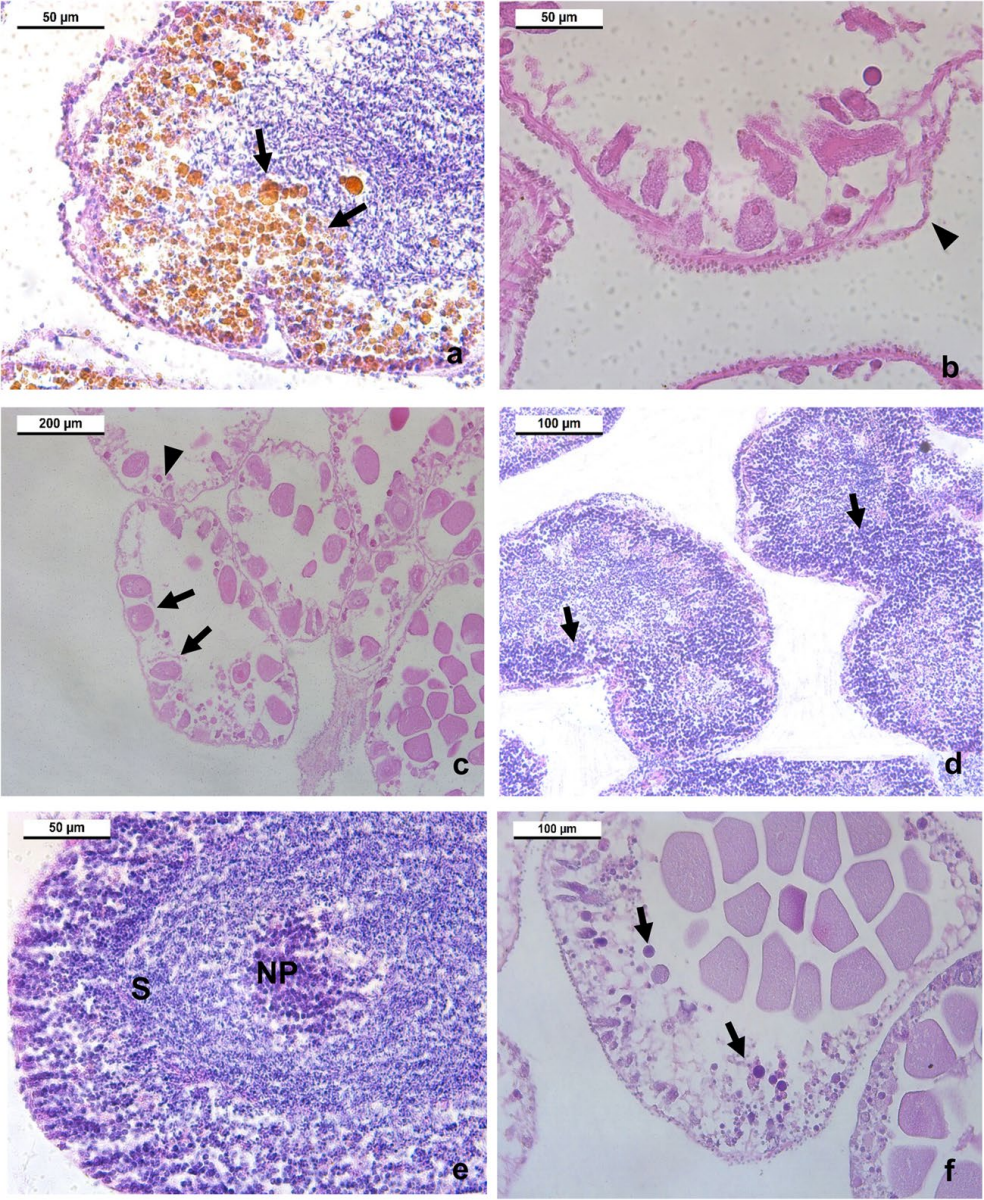
Gonadal lesions on *Paracentrotus lividus* from the two collection areas in 2017. (**a**) Male with globular inclusions of LLP (arrows); dilations of the ascinal wall (arrow heads); (**b**) female with dilated membrane; (**c**) female with atretic oocytes (arrows) and hypertrophy of NPs (arrows heads); (**d**) male with hypertrophy of NPs (arrow); (**e**) male with large aggregations of NPs in the center; (**f**) female with hypertrophy of NPs (arrows); NP, nutritional phagocytes; LLP, lipofuscin-like pigments

The analysis of histopathological lesions showed various levels of expression of pathological changes in ovaries and testes of *P. lividus* from the two different sampling stations during July, August, and September of 2017. Resorption of oocytes (IHPA reabs), dilation of ascinal wall (IHPA dilat), the presence of LLP (IHPA LLP) in different gonadal compartments, hypertrophy of NPs (IHPA hyper), or atrophy of NPs (IHPA atro), were observed in at least one individual of each sampling station and each sampling moment analyzed (Fig. 6).

Three-way ANOVA analysis showed that the gender of the individuals influenced the resorption of oocytes (IHPA reabs), presence of LLP (IHPA LLP), and atrophy of NPs (IHPA atro) found in this study. In general, female sea urchins showed a tendency to present significantly higher resorption of oocytes (IHPA reabs) and atrophy of NPs (IHPA atro), compared to males. Contrary to this trend, the presence of LLP (IHPA LLP) appears with a higher incidence in male individuals, compared to female individuals (Table 1).

The dilation of ascinal wall (IHPA dilat), the resorption of oocytes (IHPA reabs), the presence of LLP (IHPA LLP) and hypertrophy of NPs (IHPA hyper) showed significant differences over time (Table 1). All these indices followed the same pattern, being more evident in July and less evident in September. Comparisons performed using *Tukey HSD tests* showed that there were significant differences in dilation of ascinal wall (IHPA dilat), the presence of LLP (IHPA LLP), and hypertrophy of NPs (IHPA hyper) between the months of July and August, and July and September (*p =* 0.000; *p =* 0.006*; p* = 0.023*,* respectively). In contrast, the resorption of oocytes (IHPA reabs), found in the gonads of sea urchins differed statistically between the months of July and August, August and September, and July and September (*Tukey HSD test; p* = 0.000*).*

It was not detectable a particular pattern of variation on IHPA lesions between the sampling stations during the study period by the ANOVA procedures, except for dilation of ascinal wall (IHPA dilat) (Table 1). During all the months of study, and especially in July, the month in which the oil spill occurred, statistically significant differences were observed in the dilation of ascinal wall (IHPA dilat) of the gonads, with higher values in the IS (July: 1.63; August: 0.50; September: 0.30) than in the RS (July: 0.90; August: 0.43; September: 0.71) (Table 2).

**Table 2:** Index of histopathological alterations (IHPA) in the gonads of *Paracentrotus lividus* collected in the different sampling areas: impacted shore (IS) and reference shore (RS) (Peniche, Portugal) in July, August, and September of 2017, after an oil spill event

Month	Sampling station	Sex	IHPA dilat	IHPA reabs	IHPA llp	IHPA hyper	IHPA atro
July	IS	M	0.93	1.03	0.57	1.30	0.27
		F	0.70	0.77	0.20	0.47	0.50
	RS	M	0.40	0.63	0.50	0.70	0.00
		F	0.50	0.73	0.43	0.73	0.20
August	IS	M	0.37	0.47	0.33	0.63	0.07
		F	0.13	0.50	0.17	0.70	0.50
	RS	M	0.23	0.17	0.43	0.33	0.07
		F	0.20	0.63	0.13	0.43	0.67
September	IS	M	0.20	0.07	0.07	0.70	0.10
		F	0.10	0.00	0.07	0.60	0.20
	RS	M	0.07	0.00	0.40	0.60	0.17
		F	0.10	0.03	0.10	0.73	0.30

*IHPA dilat* index of membrane dilation, *IHPA reabs* index of sex cell resorption, *IHPA llp* index of lipofuscin-like pigments accumulation, *IHPA hyper* index of hypertrophy in nutritive phagocytes, *IHPA atro* index of atrophy in nutritive phagocytes

#### The influence of metals on the biometric and histopathological lesions

In the context of the histological lesions, Cd showed a positive correlation with three of the five lesions studied in this work: dilation of ascinal wall (IHPA dilation), atrophy of NPs (IHPA atrophy), and resorption of oocytes (IHPA reabs) (Table 3). Cd was positively and weakly correlated with the first two indexes and showed a positive but moderate relationship with IHPA reabs. Similarly, a weak positive correlation was observed between Cu with IHPA reabs. Zn, in turn, exhibited a poorly negative correlation with IHPA LLP, just as Fe exhibited a weakly negative correlation with IHPA reabs and IHPA hyper. Lastly, the metal Mn was negatively and weakly correlated with IHPA hyper.

**Table 3:** Spearman correlation matrix (*ρ* spearman) for metal concentrations [Cd], [Pb], [Ni], [Zn], [Cu], [Fe], and [Mn], and the index of histopathological lesions in gonads of *Paracentrotus lividus* during the study period

	IHPA dilat	IHPA reabs	IHPA llp	IHPA hyper	IHPA atro	[Cd]	[Pb]	[Ni]	[Zn]	[Cu]	[Fe]	[Mn]
IHPA dilat	1.0											
IHPA reabs	**0.546**	1.0										
IHPA llp	**0.249**	0.179	1.0									
IHPA hyper	**0.283**	0.115	−0.002	1.0								
IHPA atro	−0.004	0.137	0.046	**−0.523**	1.0							
[Cd]	**0.346**	**0.406**	0.155	−0.074	**0.288**	1.0						
[Pb]	0.002	0.150	−0.018	0.004	−0.106	0.092	1.0					
[Ni]	−0.064	0.046	−0.022	−0.031	−0.092	0.192	0.125	1.0				
[Zn]	−0.083	−0.030	**−0.218**	−0.099	0.160	**0.353**	0.007	−0.138	1.0			
[Cu]	0.094	**0.259**	0.042	0.081	−0.024	**0.260**	0.139	**0.306**	−0.016	1.0		
[Fe]	−0.188	**−0.275**	−0.037	**−0.217**	0.077	−0.092	0.064	0.128	−0.190	0.034	1.0	
[Mn]	−0.069	0.017	0.051	**−0.281**	0.135	0.037	**0.224**	0.182	−0.129	0.146	**0.611**	1.0

Significant correlations with *p* < 0.05 are highlighted in bold (*N* = 96)

*IHPA dilat* index of membrane dilation, *IHPA reabs* index of sex cell resorption, *IHPA llp* index of lipofuscin-like pigments accumulation, *IHPA hyper* index of hypertrophy in nutritive phagocytes, *IHPA atro* index of atrophy in nutritive phagocytes

Regarding the biometric variables, the GW and the GSI of sea urchins were negatively and weakly correlated with Cd (Table 4). The GSI also exhibited a positive but only weak correlation with Fe. The WW of the sea urchins was negatively and moderately correlated with the concentrations of Pb, while a negative and weak correlation was found with the metal Ni. The metal Cu showed a weak and negative correlation with the individual’s DC. As expected, the GW and the GSI were positively and strongly correlated with each other. The DC of the animals showed a moderate correlation with the weight of the gonad; in turn, the weight of the gonad showed a positive, albeit weak, correlation with the individual’s total weight. In addition, significant positive correlations were also observed between different metals: Zn and Cd, Cd and Cu, Pb and Mn, Pb and Ni, and lastly, the strongest among all observed, Fe and Mn.

**Table 4:** Spearman correlation matrix (*ρ* spearman) for metal concentrations [Cd], [Pb], [Ni], [Zn], [Cu], [Fe], and [Mn], and biometric variables in gonads of *Paracentrotus lividus*, namely the diameter of the carapace (DC), total wet weight (WW), gonadal weight (GW), and gonadosomatic index (GSI)

	DC	WW	GW	GSI	[Cd]	[Pb]	[Ni]	[Zn]	[Cu]	[Fe]	[Mn]
DC	1.0										
WW	**0.471**	1.0									
GW	**0.528**	**0.277**	1.0								
GSI	−0.003	−0.035	**0.765**	1.0							
[Cd]	−0.069	0.069	**−0.225**	**−0.236**	1.0						
[Pb]	−0.175	**−0.441**	−0.081	0.010	0.092	1.0					
[Ni]	−0.186	**−0.015**	−0.166	−0.092	0.192	0.125	1.0				
[Zn]	0.104	0.043	0.020	−0.026	**0.353**	0.007	−0.138	1.0			
[Cu]	**−0.207**	−0.144	−0.195	−0.076	**0.260**	0.139	**0.306**	−0.016	1.0		
[Fe]	−0.119	−0.106	0.106	**0.243**	−0.092	0.064	0.128	−0.190	0.034	1.0	
[Mn]	−0.130	−0.132	0.003	0.120	0.037	**0.224**	0.182	−0.129	0.146	**0.611**	1.0

Significant correlations with *p* < 0.05 are highlighted in bold (*N* = 96)

## Discussion

Sea urchins are recognized to be very sensitive to changes in environmental conditions. This sensitivity is manifested by disparities in the reproductive cycle, decline in fertility, and developmental disorders (Auernheimer and Chinchon, 1997; Savriama et al., 2015). These disorders affect the growth and physiology of these echinoderms; thus, changes in biometric parameters and biological indices in this species are integrated indicators that may reflect the effects of pollution and environmental quality of a given area, especially those exposed during the development of these animals (Warnau et al., 1997; Chiarelli and Roccheri, 2014; Ouchene et al. 2021).

The monitoring of the metal contents in *P. lividus* gonads showed temporal and spatial variations according to each one of the metals analyzed. These variations may be closely associated with the availability of metals in the environment, the physiological changes on the echinoderms and the environmental disturbances related to the quality of the environment in both sampling stations (Filipuci 2011; Rouane-Hacene et al. 2018; De Zoysa et al. 2018; Ouchene et al. 2021). Due to their predominantly herbivorous feeding habits, sea urchins can absorb metals dissolved in seawater, metals contained in the diet — especially algae — and presumably also metals bound to particles which are ingested. Bioaccumulation of metals in sea urchins can therefore be considered a bridge to uptake of metals from these sources (Søndergaard et al. 2019).

In this study, the bioaccumulated metals detected in the gonads of *P. lividus* varied according to the following pattern: Fe> Zn> Ni> Cu> Mn> Pb> Cd. In both sampling sites, Zn was the most abundant element in the gonads of *P. lividus*, while Cd was always the scarcest one, indicating that *P. lividus* has a greater tendency to accumulate essential metals such as Zn and Fe. On the other hand, the concentrations of toxic metals as Cd, Pb, and Cu in the sea urchins analyzed in the present study were less expressive or very similar to those reported by other authors who developed work on the European coast (Warnau et al. 1995a; Soualilli et al. 2008; Strogyloudi et al. 2014; Bouiba et al. 2023). In accordance with our results, these comparisons do not consider data related to whole organisms, but only the gonads of the animals. The concentration and behavior of metals are closely associated with the physiological state of the organism and the phases of their reproduction cycles (Hernández et al. 2010).

In studies using *P. lividus* as a biological indicator, Warnau et al. (1998) reinforces the need to consider different body compartments and the sampling period as important factors. Variations in metal concentrations can be greater between different body compartments than between areas. In view of this, comparisons between the levels of metals should only be carried out in compartments and/or tissues collected at the same time of year. In sea urchins, among the compartments seen as good useful tools for biomonitoring, the digestive wall, the gonads, and the body wall stand out (these compartments had the highest concentrations of metals). Although, it is important to highlight that the use of gonads should be avoided during the spawning period, as the occurrence of metal loss through gamete release can lead to incorrect comparisons between different echinoid populations (Warnau et al. 1998). However, in the present study, since there was mostly a synchronization in the gametogenic cycle between the two populations under study, it does not preclude the comparison between populations, as it will not be subjected to this effect.

The results of this work also demonstrated that Zn and Cd concentrations were clearly and significantly higher in the gonads of females, at both the temporal and spatial scales. According to Watling and Watling (1976), Zn is an essential element for reproduction in several marine organisms. Also, high concentrations of Zn in female gonads relative to other tissues during the reproductive period are common in many marine invertebrates (Orren et al., 1980; Ahn et al. 2002). Despite that, sea urchin sperm also contains considerable amounts of Zn, compared to Cu and Mn, which means that spermatogenesis also requires large amounts of Zn, although the demand for spermatogenesis is lower than for oogenesis. In male animals, including sea urchins, Zn is essential for sperm motility and for the acrosome reaction (Clapper et al., 1985). Studies suggest that the primary purpose of Zn to the ovary and testis is to provide essential supplies for oogenesis and spermatogenesis (Unuma et al., 2007).

The interaction between the factors sexes, rocky shores, and months had a significant effect on the mean Cd values observed in *P. lividus* gonads. According to Jakimska et al. (2011), the presence of the metal Cd in animal tissues may indicate both short and long-term exposures, since absorption of this element is not controlled by active homeostasis. Usually, Cd levels in internal tissues such as gonads are also related to the typically high metabolic activity of these organs and tend to increase during the reproductive cycle, but this was not observed in our study contrarily to the reported by some authors (Warnau et al. 1995a, b; Den Besten et al. 2001). On the other hand, the results for Cd concentrations found in this study, which differed statistically between males and females, are in agreement with the findings of Soualli et al. (2008) for *P. lividus* from Algiers (Algeria). These authors determined the concentrations of various metals, such as Zn and Cd, in sediments and sea urchin gonads, and found that the most numerous larval abnormalities were observed in a location near Algiers, identified as highly polluted by Pb. Levels of the other metals, including Cd and Zn, were significantly higher in female gonads than in male gonads.

Ouchene et al. (2021) developed a study similar to ours, in which seasonal variations in the gonadal index (GI), biochemical composition, and concentration of trace metals in *P. lividus* gonads were determined. The organisms were collected from three sites in the Agadir region: Cap Ghir, Sidi R’bat, and Anza (southern coast of Morocco) between March 2018 and February 2020. The results of the study indicated that trace metals Cu, Pb, and Cd were present with very low values, and this reflects the health of the coastal ecosystem of Cap Ghir and Sidi R’bat. On the other hand, for the locality of Anza, higher values than the other sites were observed, due to the presence of industries and urban development. A similar context was observed in the present study. The comparison of the concentrations of trace elements analyzed in the gonads of *P. lividus* with those from other regions of the world shows that the concentrations of trace metals in our samples are relatively low, but Cu and Pb were present in high concentrations, especially in RS.

Some studies have investigated spatial variations in element concentrations near sources of anthropogenic pollution in *P. lividus* and other sea urchin species such as *Strongylocentrotus droebachiensis* and *Lytechinus variegatus*. In these studies, high concentrations of several elements were observed near polluting sources, and these species were proposed as biomonitors for several elements, like for instance Fe, Cu, Zn, Cd, and Pb (Scanu et al. 2015; Alves et al. 2018; Rouane-Hacene et al. 2018; Ternengo et al. 2018; Søndergaard et al. 2019; Ouchene et al. 2021).

Complementarily, our study showed that the concentrations of Pb exhibited statistical differences between the rocky shores analyzed, being, in most cases, higher at RS in relation to the IS. Sany et al. (2013) affirms that chemical properties of metals, water, and sediment are associated with other environmental factors such as physical variables of the external environment (atmospheric deposition, high seawater dynamics, changes in currents, and anthropogenic pollution load shifting); changes in these factors may affect the solubility and distribution of metals in the environment and sediments which consequently affect the toxicity of the metal in marine organisms. The dissipation of metals in animal tissues depends on the duration of exposure and the concentration of the element in the immediate environment. In addition, these differences may also be associated with the reduction or increase (depending on the month) of anthropogenic activities on the coast during this period, since from July to September the visit to The Berlengas Archipelago (an UNESCO Biosphere Reserve) is allowed. In July and August, boat traffic on the west side of the Peniche peninsula is intense, decreasing again in September, when the access to the Archipelago closes again. In addition, Pb may be more present in the water when the months of more intense rainfall begin, characteristic of autumn, which in Portugal begins at the end of September. This possible increase in Pb concentration may be associated with increased precipitation. Contamination can be direct by atmospheric deposition and indirect by leaching that occurs on roads caused by rainwater (Ouchene et al. 2021).

Within the scope of biometric parameters, the population from the IS presented significantly higher DC and WW values, compared to individuals collected at the RS. These values varied significantly between the gender of the individuals collected, the rocky shores, and over the 3 months of sampling (July, August, and September). These patterns of spatial and temporal variability can be better understood considering in addition to aspects of the sea urchin reproductive cycle progress, environmental, and ecological factors fundamental to the maintenance and distribution of these organisms and to their gametogenic cycle.

The variability of biometric parameters and somatic growth of sea urchin populations is strongly influenced by factors such as local hydrodynamics (Siddon and Witman 2003; Micheli et al. 2005; Bertocci et al. 2014), food availability and quality, and gonadal development (Boudouresque and Verlaque 2001). Turon et al. (1995) state that in a habitat exposed to low quality food, the maximum growth and size of *P. lividus* is generally lower than in a deeper habitat where food supply is not limited. The presence of smaller individuals in a given area, therefore, may be driven by adverse conditions that these populations face in terms of high-water dynamism and intermittent food availability. In fact, RS is a rocky shore clearly more exposed to the hydrodynamic impact of the waves, compared to the IS, which has a bay-shaped rocky coast and a low hydrodynamic impact. Wave exposure and hydrodynamics may influence algae abundance and, therefore, the availability and quality of food for the sea urchins inhabiting these two areas. Within this context, the DC and WW values of sea urchins at the RS are possibly lower due to the great disturbance suffered by the organisms, requiring physiological and morphological adaptations, directing energy for the development of efficient protection, fixation, and reproduction structures to withstand environmental stress (Satyam and Thiruchitrambalam 2018); altogether this may explain why the sea urchins at RS were significantly smaller and lighter than the animals from the IS.

Although the animals were significantly larger and heavier in the IS, the GSI values observed in this area (7.49 ± 0.72%) were lower than the values recorded in the RS population (9.50 ± 0.28%). This finding agrees with those obtained by Turon et al. (1995) in north-eastern Spain, which detected higher somatic growth in habitats characterized by low wave exposure. As in this study, the energy invested in reproduction followed the inverse pattern, and gonad production was higher in a changing habitat subjected to strong wave action (Lozano et al. 1995). This explanation may also justify the fact that the average DC of the IS population is larger than at the RS. Sea urchins inhabiting the RS zone are exposed to the action of high waves, so they may favour reproduction over somatic growth in stressful situations.

Some compartments of marine organisms can accumulate trace metals as soon as in contact with toxic concentrations. This condition can trigger reduction-oxidation reactions generating free radicals and, consequently, cause biochemical and morphological modulations (Varanka et al. 2001; Monteiro et al. 2005). Cd levels showed a significant negative correlation with gonadal weight and GSI, demonstrating that as Cd accumulation increases, the gonadal weight and the gonadosomatic index of these sea urchins tend to decrease. Cd is a highly toxic metal whose accumulation in the gonads can have serious implications, not only for species whose gonads are commercially harvested for human consumption, but also concerning the production of non-viable gametes in these echinoids (Järup et al. 1998; Chiarelli and Roccheri 2014). In sea urchins, Cd levels in internal tissues such as gonads are also related to the typically high metabolic activity of these organs and tend to increase during the reproductive cycle (Warnau et al. 1995a, b; Den Besten et al. 2001), a trend that was not observed in this study. In general, Cd exhibits a relatively long biological half-life in sea urchins: more than 70% of the Cd absorbed by the echinoids was slowly eliminated, in approximately 1 year in the study developed by Warnau et al. (1997).

The WW and the DC correlated negatively and significantly with Pb and Cu, respectively. Pb, particularly, accumulates in the bodies of aquatic organisms and of organisms that live in the soils and is a bio-persistent pollutant that accumulates at the top of the food chain. Moreover, Pb-induced toxicity to marine invertebrates varies according to species and their life stage (Guillou et al. 1995; Auernheimer and Chinchon 1997; Rouane-Hacene et al. 2018; Chiarelli et al. 2019). Despite the differences between the two sampling stations, the biometric variables evaluated in this study followed similar variation patterns in both IS and RS over time. Indeed, in most cases, the lowest average values of biometric variables were recorded in August and, in contrast, the highest average values were observed in September. In addition to being influenced by all the factors abovementioned, this pattern also reflects the events that occur during the process of gametogenesis, in which sea urchin gonads grow and/or increase the number and size of germ cells in two distinct periods: before gametogenesis (when nutritional reserves are stored in nutritional phagocytes) and during gametogenesis (when the number and the size of gametes are larger).

**Fig. 7:**
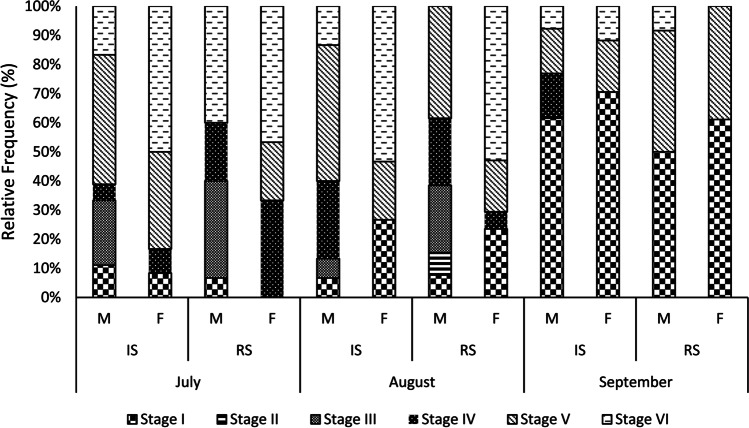
Variation on the gametogenic stages of *Paracentrotus lividus* at the impacted shore (IS) and at the reference shore (RS) (Peniche, Portugal) collected in July, August, and September of 2017, after an oil spill event. I — initial; II — growth; III — premature; IV — matures; V — posture; VI — post-posture

The gametogenic cycle observed in *P. lividus* specimens collected followed a recognizable pattern, characterized by six gametogenic stages (Byrne 1990). Due to the reduced time to collect the sea urchins, it was observed an incomplete reproductive cycle. Nevertheless, a comparison was made between the results of this study in the months of July, August, and September, in contrast to other studies performed. Histological analyses showed that sea urchins from both rocky shores were at a similar stage of the gametogenic cycle as the population sampled at Abalo Beach between 2015 and 2016 (Raposo et al. 2019). However, in July, the results seem to indicate a slight delay in the reproductive cycle of the sea urchins at IS, as those were mostly in stage V, compared to the RS, and had fewer individuals in stages III and IV. This delay may be related to high concentrations of Pb found in sea urchins from the IS, which could also be responsible for the high indices of IHPA dilat in July. Although, and to our knowledge, there are no published studies confirming the influence of trace metals on the delay of the gametogenic cycle of echinoderms; there is already proven evidence that this happens in other marine species (Thomas 1988; Gauthier-Clerc et al. 2002; Siah et al. 2003). Nevertheless, the difference in the gametogenic cycle between the two sites seems to have become less evident over the remaining months, suggesting that the sea urchins at the IS site have managed to recover from the gametogenic delay (Fig. 7).

Using a histological parameter, it was possible to identify dilation of acinal wall, resorption, hypertrophy and atrophy in nutritive phagocytes, and marked accumulation of lipofuscin-like pigment accumulation, as previously described by Vaschenko et al. (2001), in individuals from both sampling stations during the 3 months of study. A histopathological study developed by Schäfer et al. (2011) with *Psammechinus miliaris* showed that after exposure to a sublethal concentration of phenanthrene — a polycyclic aromatic hydrocarbon present in the oil, known as a severe ovarian lesions agent — and, as noticed in the present work, the size of the gonad and the gonad index of sea urchins was significantly reduced, designating atrophy.

Although high IHPA LLP were found in both sampling stations in July, it could be associated not only to the toxic spill but also to the gametogenic cycle stage, the sea urchins were in (Miranda et al. 1999; Vaschenko et al. 2012). Normally, in the post-spawning stage of the gametogenic cycle or under unfavorable environmental conditions, such as temperature change and presence of pollutants, the resorption processes characteristic of this stage induce the accumulation of yellow pigments in the NP cytoplasm or in different compartments of the gonad (Vaschenko et al. 2012). Indeed, the lipofuscin content in sea urchin gonads is closely associated with the final reproductive stage of the animals, with the highest levels found in partially spawned and spawned individuals (Schäfer et al. 2011), as shown in the present work. The final gametogenic stages can also explain the resorption and dilation rates of the acinus membrane. Although several studies address this issue in other marine invertebrates (e.g. bivalves), only a few studies have investigated the effects of chemical pollutants on gonadal tissue of sea urchins. In this way, our work demonstrates the relevance of investigating the effects of trace metal exposure on sea urchin gonads using histopathological changes as biomarkers. Therefore, although this work did not cover the complete reproductive cycle of *P. lividus* and analyses were not carried out to determine PAH concentrations, the finding of contaminants such as Cd in the gonads of these animals suggests that the delay in the gametogenic cycle observed in sea urchins at the IS, may be related to direct exposure to oil and its components during the spill and to the higher incidence of lesions observed in the gonads of these animals.

Over time, the IHPA dilat, IHPA reabs, IHPA LLP, and IHPA hyper varied significantly. All these indexes followed the same pattern, showing higher values in July and decreasing until September. These findings indicate that probably in the final process of gametogenesis the lipofuscin was developed, accumulated throughout the process, and released from the gonads (Miranda et al. 1999). Otherwise, larger animals that had already undergone gametogenesis in previous years would have a higher lipofuscin content than the smaller and younger ones. In this study, the *P. lividus* population from the IS presented significantly higher diameter of the carapace (DC) and total wet weigh (WW) values, compared to individuals collected at the RS. As lipofuscin levels decrease after spawning, it was assumed that lipofuscin is released during the recovery period. This reasoning could explain the pattern observed at the IS not only for lipofuscin, but also for the resorption, hypertrophy and dilation rates of the acinus membrane.

The presence of contaminating metals in the gonads of *P. lividus* at both sampling sites seemed to affect the gonads and triggered the histopathological disturbances, which was corroborated with the correlation analyses. As expected, since Cd is a highly toxic metal, this element exhibited positive correlations with the IHPA resorption, dilation, and LLP. Migliaccio et al. (2015) evaluated the effects of Cd and Mn in adults of *P. lividus* and their offspring and observed that both metals differentially impair the fertilization process of treated sea urchins, causing changes in reproductive status and consequently generating abnormal embryos. Associated to this, the increasing nitric oxide production in the ovaries triggers variations in transcriptional expression of various genes involved in stress response, skogenesis, detoxification, and multiple drug efflux processes. As reported, although Cu is an essential metal for all eukaryotic organisms, it can reach toxic levels in aquatic environments entailing also reactive oxygen species may be formed, causing cytotoxicity and DNA damage (Bryan and Langston 1992; Stohs and Bagchi 1995; Zorita et al. 2006).

The levels of Zn, Fe, and Mn elements correlated negatively with IHPAs, LLP, resorption, and hypertrophy, respectively, suggesting that these essential metals play an important role in curing these lesions, since as their gonad content increases the histopathological indices decrease. In fact, Zn in particular, is essential for cell proliferation and differentiation and is a prerequisite for chromatin structure (Vallee and Auld 1990; Coleman 1992).

## Conclusion

Toxic compounds from oil spills, such as trace metals, can pose a serious threat to marine invertebrates in coastal areas. Sea urchins, in particular, are susceptible to contact with these pollutants, since they live attached to the substrate and have limited locomotion. The exposure of these adult animals to adverse environmental conditions can cause changes in their behavior and physiology; however, studies using these organisms in adulthood as environmental assessment tools are still scarce. In this study, the occurrence of slight effects in the reproductive function of *P. lividus*, due to high incidence of histopathological lesions, which may be related to direct exposure to contaminants during the spill, were demonstrated. With the results obtained, it was possible to observe that the exposure of *P. lividus* to contaminants from oil spills, such as trace metals, resulted in harmful effects at a reproductive and physiological level for these animals. Thus, this study proposes the assessment of the gonads of the sea urchin species *Paracentrotus lividus* in environments exposed in situ to trace metals from oil spill as bioindicator of contamination by pollutants, since the responses observed in this species may have useful application as an environmental impact assessment tool in marine environments. Studies including bioindicators present an opportunity for a more meaningful way of assessing the effects of pollution on ecosystems. Lastly, it is possible to understand the sensitivity of environments to changes, the resulting impacts on biodiversity and the risks to public health directly or indirectly related to environmental quality in those areas. This paper provides evidence of the physiological and histological consequences that trace metals can generate in adult sea urchins and contributes to the understanding of the effects of exposure to trace metals derived from oil spills in these echinoderms.

## Data Availability

Not applicable
